# Analysis of Possible Intermediate Hosts of the New Coronavirus SARS-CoV-2

**DOI:** 10.3389/fvets.2020.00379

**Published:** 2020-06-09

**Authors:** Shu Yuan, Si-Cong Jiang, Zi-Lin Li

**Affiliations:** ^1^College of Resources, Sichuan Agricultural University, Chengdu, China; ^2^Chengdu Kanghong Pharmaceutical Group Co., Ltd., Chengdu, China; ^3^Department of Cardiovascular Surgery, Xijing Hospital, Medical University of the Air Force, Xi'an, China

**Keywords:** coronavirus, ecological niche, relative synonymous codon usage, rodent, SARS-CoV-2

Since infections with the new pneumonia virus (SARS-CoV-2) were first reported in China, the epidemic has spread rapidly. Now the virus has spread beyond China, and international exportation into most countries in the world is occurring. To date, the source(s) and complete route of transmission of the virus have not been clarified.

## SARS-CoV-2 is a Bat-Derived Betacoronavirus

**Figure 1:**
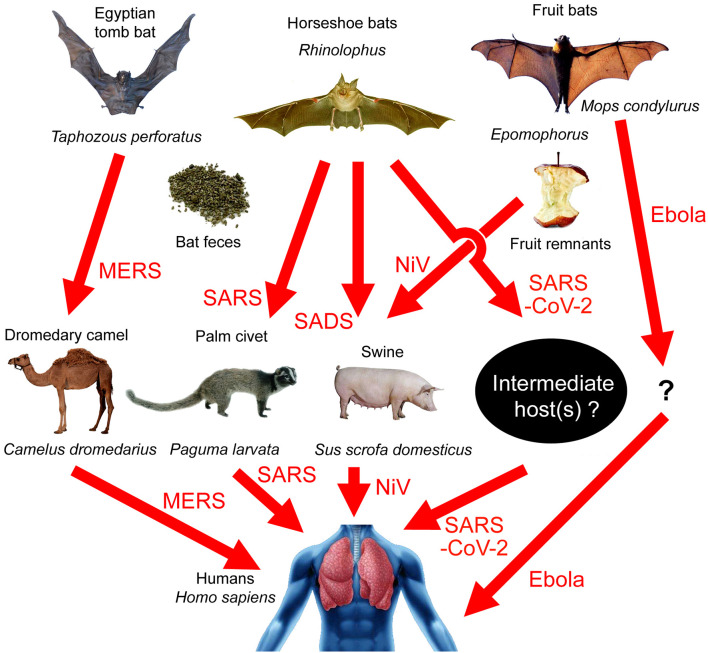
Transmission paths of bat-borne viral diseases. MERS: Egyptian tomb bat → dromedary camel → humans; SARS: horseshoe bats → palm civets → humans; SADS: horseshoe bats → swine; SARS-CoV-2: horseshoe bats → unknown intermediate host → humans; Nipah virus (NiV): fruit bats → swine → humans; Ebola virus: Angora dog bat (*Mops condylurus*) → unknown intermediate host → humans.

Four recent articles analyzed the whole-genome sequence of SARS-CoV-2 and constructed phylogenetic trees (1–4). It is believed that the virus belongs to the *betacoronavirus* genus, and the SARS-CoV-2 cluster is situated with the groups of SARS (Severe Acute Respiratory Syndromes)/SARS-like coronaviruses, with fruit bat coronavirus HKU9-1 as the immediate outgroup (2). A recent study pointed out that the similarity between SARS-CoV-2 and BatCoV RaTG13, a virus strain isolated from *Rhinolophus yunnanensis*, was as high as 96.2% (3). Bats are therefore the most probable source of the virus. Bats can carry many kinds of viruses without becoming ill in response to them (5, 6). There is a huge natural coronavirus pool in bats that sometimes spreads to humans. For example, the Ebola virus originated from the Angora dog bat (*Mops condylurus*, a fruit-eating bat), although its intermediate host is still unknown (7). The MERS (Middle East Respiratory Syndrome) virus originated from the Egyptian tomb bat (*Taphozous perforatus*) and was transmitted to the dromedary camel (*Camelus dromedarius*) before going on to infect humans [Figure 1; (8–12)].

## Snakes and Birds May Not be the Intermediate Hosts

Fruit bats are rarely found in Hubei province, but horseshoe bats (*Rhinolophus*) are widely distributed there and are likely to be the source of the virus. But how does *Rhinolophus* spread SARS-CoV-2 to humans? Some intermediate host(s) may be involved. A recent study suggests that SARS-CoV-2 may be derived from the homologous recombination of a bat coronavirus with a snake coronavirus (13). They compared the relative synonymous codon usage (RSCU) bias of the viral genome with its possible hosts and found that the RSCU bias of SARS-CoV-2 is most close to that of snakes (13). However, in that study, the numbers of codons of the two snakes were several orders of magnitude lower than those of other species. This kind of comparison may be inappropriate. In addition, betacoronavirus has never been detected in snakes (14). The host range of a certain coronavirus is relatively narrow. For example, the SARS-like coronavirus reported in *Rhinolophus hupehensis* during 2005 could not infect human cells (15). The authors of the above report continuously searched bat coronaviruses in China for the subsequent 8 years until, in 2013, they found a SARS-like coronavirus isolate WIV1 in Yunnan province that could infect cells from both humans and other mammals (16). Even if a super-highly contagious betacoronavirus strain emerges, it may not cross over mammals to infect snakes, tortoises (17), or birds. Furthermore, wild snakes were in hibernation in winter and are unlikely to act as the intermediate hosts.

## Palm Civet and Other Carnivorous Animals Are Unlikely to be the Intermediate Hosts

Studies on SARS indicated that the palm civet (*Paguma larvata*) may be an intermediate host. In fact, before the outbreak of SARS, civet feeding became popular in many parts of China. Bat SARS-like coronavirus may have infected palm civets by accident in a Yunnan civet-farm. The virus-carrying civets may then have been sold to Guangdong province. The virus may have further spread and mutated in palm civets on the market, resulting in a highly contagious SARS virus, which infected humans in 2003 [Figure 1; (18–20)]. However, in the case of SARS-CoV-2, the first patient and 12 of the 40 later cases had no link to the wild animal market in Wuhan (21, 22). Therefore, the animals usually seen in the market, such as palm civets, are unlikely to be the original intermediate hosts of the virus.

The identity of the full-length spike (S) glycoprotein between palm civet SARS coronavirus [AY515512.1; (23)] and SARS-CoV isolated from humans (AH012999.2) is 98.0%, while the S glycoprotein identity between SARS-CoV and SARS-CoV-2 (YP_009724390.1) is only 75.4%, also indicating that palm civets are the intermediate hosts of SARS-CoV but not SARS-CoV-2.

Like palm civets, other animals usually seen in the market may also be not the intermediate hosts, such as hog badgers, dog badgers, coyotes, and raccoon dogs. A recent study showed that SARS-CoV-2 replicates poorly in dogs, pigs, chickens, and ducks, but ferrets and cats are permissive to infection (24). However, cats and dogs may usually be infected with alphacoronaviruses, not betacoronavirus (14). To define the possible roles of domesticated animals in SARS-CoV-2 transmission, further investigations are required.

## Pangolin May Not be the Direct Intermediate Host

Xiao et al. (25) recently reported betacoronavirus in pangolins. However, the statement of “as close as 99%” that made in their press release for the pangolin virus was misleading because the full-length genome similarity between the pangolin virus and SARS-CoV-2 is only 90.3%. A high similarity of 99% has been found within the “E” region (25). However, viruses from other species are also very similar in this region. Lam et al. (26) also reported several genome sequences of coronaviruses isolated in Malayan pangolins. Although a high similarity of 97.4% has been found within the receptor-binding domain, the full-length genome similarities between the pangolin coronaviruses and SARS-CoV-2 (85.5–92.4%) are much lower than that between BatCoV RaTG13 and SARS-CoV-2 (96.2%). Additionally, in the phylogenetic tree, the pangolin CoV cluster is situated outside the clade of human CoV and *Rhinolophus* CoV (25, 26). Viruses from the direct intermediate host should be closer to humans than to bats. Moreover, the highest similarity between pangolin coronaviruses and SARS-CoV-2 is only 92.4% (26), indicating that there is a large genetic distance that needs decades of evolution. Andersen et al. (27) further found that neither pangolin CoV nor the BatCoV RaTG13 carries the polybasic cleavage site insertion that is required for human ACE2 receptor binding. Thus, SARS-CoV-2 does not seem to be the result of the recombination of a pangolin virus with a bat virus (28).

Moreover, China's pangolins are on the brink of extinction, and almost no wild pangolin could be caught. Such a low population density makes it almost impossible that it is an intermediate host. Lam et al. (26) suggested that pangolins should be removed from wet markets to prevent zoonotic transmission. However, pangolins have long been banned from sale, and pangolins could not be seen in the market. It would have been almost impossible for the first generation of patients to come into contact with living pangolins.

## Livestock May Not be the Intermediate Hosts

Betacoronavirus can infect *Artiodactyla* and *Perissodactyla* animals, such as swine, cattle, horses, camels, etc. In 2018, researchers identified a pathogen causing acute lethal diarrhea of piglets in a pig-farm in Guangdong, which was a new type of bat-derived Swine Acute Diarrhea Syndrome coronavirus (SADS-CoV), although the virus did not transmit to humans (29). They did indeed observe *Rhinolophus* flying around the pig-farm, and the bat feces may be the transmission media [Figure 1; (29)]. Malaysia's Nipah virus (NiV) has a similar transmission path. Local people built pig farms next to bat habitats. Bats that were carrying the virus ate fruit and dropped virus-polluted fruit parts into the pig pens. Through this route, NiV infected swine and then infected humans [Figure 1; (30)]. However, livestock infected with coronaviruses would show serious symptoms and even death, and yet there have been no recent reports of acute diseases in livestock in Wuhan, though the possibility of asymptomatic infection cannot be ruled out.

## Rodents May Play an Important Role in the Viral Transmission

During animal selection, the viral genome should make some adaptations to the host, such as changes in the relative synonymous codon usage (RSCU) bias. Ji et al. (13) interestingly indicated that, among all possible mammal hosts, the RSCU bias of SARS-CoV-2 is most close to that of *Marmota* (a rodent species), which may indicate rodents as the intermediate hosts. The two endemic human coronaviruses, HCoV-OC43 and HCoV-HKU1, have been suggested to have originated from rodents (31). Besides, young mice (4–6 weeks old) do not develop illness following SARS infections, while older (12–14 months old) mice develop clinical illness and pneumonitis but do not subsequently die (32, 33). This suggests that one or more rodent species may be the intermediate hosts in which the virus was circulating and mutating (34).

The ecological niche overlap between the city mouse and the *Rhinolophus* (active in the mountains) is low. Given that both rats and bats are widely distributed around the world, the single-point outbreak centered on Wuhan cannot be reasonably explained. Bamboo rats are widely cultured in China. However, they eat bamboo roots and stems, grass shots, and so on, which have no overlap with the ecological niche of *Rhinolophus*. We have noticed that a large number of squirrels have been released in Wuhan since 2013, and a park for wild squirrels has been built in Wuhan. Both wild squirrels and *Rhinolophus* are active in mountain forests, and their ecological niches overlap to some extent. People usually treat squirrels as pets and feed them without any protection. They might transmit SARS-CoV-2 through saliva or by accidental biting during feeding. Although no coronavirus has been isolated from squirrels so far, the lymphocytic choriomeningitis virus and some lyssaviruses have been reported in *Sciuridae* animals (35), implying a zoonotic transmission capability from squirrels.

Hamsters were found as suitable laboratory animals for SARS-CoV-2 as it causes disease and pathology in them that is somewhat close to the effects in human (36). This requires further study.

## Selection in an Animal Host vs. Cryptic Adaptation to Humans

Andersen et al. (27) proposed two theories of the origins of SARS-CoV-2: selection in an animal host or cryptic adaptation to humans. It is possible that a progenitor to SARS-CoV-2 jumped from a non-human animal (bat or some intermediate host) to humans (directly or indirectly), with its genomic features (like the polybasic cleavage site and O-linked glycans) acquired through adaptation during subsequent human-to-human transmission. However, cryptic adaptation in humans (for example, that of seasonal influenza) should result in widespread outbreaks, not a single-point outbreak. Furthermore, there should be a lot of intermediate types of viruses between the progenitor virus and the current SARS-CoV-2, whereas there are only 120 substitution sites (0.41%) found in eight coding sequences of the SARS-CoV-2 viral genome. The genomic variation of SARS-CoV-2 is still very low, and no intermediate types such as are proposed above have been found (37).

Putting aside the human adaptation theory, the animal selection theory suggests that SARS-Cov-2 have been circulating in one or more animal species before human infection. For a precursor virus to acquire the genomic features suitable for human ACE2 receptor binding, an animal host would likely have to have a high population density to allow natural selection to proceed efficiently (27). It is interesting to note that rodent betacoronaviruses have the polybasic cleavage site (38). Considering the above, surveillance and whole genomic analysis of CoVs from rodents are important to elucidate whether these species have any role in the transmission cycle of the virus and to detect the emergence of possible recombinants involving CoVs from these species and those from bats. However, there is not yet any evidence on the role of rodents or squirrels as intermediate hosts.

## Author Contributions

SY conceptualized the analysis and wrote the original draft. S-CJ and Z-LL reviewed and edited the manuscript. All authors have read and agreed to the published version of the manuscript.

## Conflict of Interest

S-CJ was employed by the Chengdu Kanghong Pharmaceutical Group Co., Ltd. The remaining authors declare that the research was conducted in the absence of any commercial or financial relationships that could be construed as a potential conflict of interest.
